# Perinatal sleep disruption and postpartum psychosis in bipolar disorder: findings from the UK BDRN Pregnancy Study

**DOI:** 10.1016/j.jad.2023.11.005

**Published:** 2023-11-07

**Authors:** A. Perry, K. Gordon-Smith, K.J.S Lewis, A. Di Florio, N. Craddock, L. Jones, I. Jones

**Affiliations:** 1 Psychological Medicine, University of Worcester, UK; 2 Division of Psychological Medicine and Clinical Neurosciences, School of Medicine, Cardiff University, UK; 3 Department of Psychiatry, Brigham & Women’s Hospital, Harvard Medical School, Boston, MA 02115

**Keywords:** Bipolar disorder, sleep, postpartum psychosis, aetiology, triggers

## Abstract

**Background:**

Women with bipolar disorder (BD) are at high risk of postpartum psychosis (PP). The factors that increase risk of PP among women with BD are not fully understood. Here, we examine whether sleep disruption in the perinatal period (poor sleep quality in late pregnancy and sleep deprivation related to childbirth) is associated with PP in a longitudinal study of pregnant women with BD.

**Methods:**

Participants were 76 pregnant women with lifetime DSM-5 bipolar I disorder or schizoaffective-BD, followed from week 12 of pregnancy to 12 weeks postpartum. Demographics and lifetime psychopathology were assessed at baseline via semi-structured interview (Schedules for Clinical Assessment in Neuropsychiatry). Psychopathology and sleep disruption within the current perinatal period were assessed in the third trimester and at 12 weeks postpartum. Data were supplemented by clinician questionnaires and case-note review.

**Results:**

After controlling for prophylactic use of mood stabilising medication, the loss of at least one complete night of sleep across labour/delivery was associated with five times the odds of experiencing PP compared to no or less than one night of sleep loss across labour/delivery (OR 5.19, 95% CI 1.45-18.54; p=0.011). Sleep quality in late pregnancy was not associated with PP, and perinatal sleep disruption was not associated with postpartum depression.

**Limitations:**

Lack of objective measures of sleep factors.

**Conclusions:**

In the context of other aetiological factors, severe sleep loss associated with childbirth/the immediate postpartum may act as a final trigger of PP. These findings could have important clinical implications for risk prediction and prevention of PP.

## Introduction

Women who have bipolar disorder (BD) are at high risk of recurrence of mood episodes in relation to childbirth, with between 37-50% estimated to be affected in the postpartum period (Di Florio et al., 2013; Wesseloo et al., 2016). Episodes of postpartum non-psychotic depression (PPD) are common in women with BD, occurring in 19-60% of deliveries (Di Florio et al., 2015; Mandelli et al., 2016; Viguera et al., 2011). However, compared to other times in a woman’s life, the risk of mania or affective psychosis (consistent with the concept of postpartum psychosis; PP) is especially high soon after childbirth (Munk-Olsen et al., 2009). It is estimated as many as one in five women with BD experience PP (Perry et al., 2021a; Wesseloo et al., 2016), placing these women at dramatically increased risk compared to women with other psychiatric disorders (Langan Martin et al., 2016) and also to the general population, in which PP occurs in 1-2 in every 1000 deliveries (VanderKruik et al., 2017). Women who have bipolar I disorder (BD-I) or schizoaffective disorder bipolar type (SA-BD) are particularly vulnerable to PP (Di Florio et al., 2018, 2013; Maina et al., 2014; Perry et al., 2021a).

PP is a psychiatric emergency, which can have devastating consequences for the mother, her baby and wider family (Brockington, 2017; Gressier et al., 2017; Hoffman et al., 2017; Holford et al., 2018). Understanding aetiological and triggering factors is therefore crucial for risk prediction and management of these episodes. The precise mechanisms involved in the pathogenesis of PP remain uncertain, though genetic, hormonal and immunological factors have all been indicated to play a role (Perry et al., 2021b). Conversely, the evidence for the importance of psychosocial factors is less compelling (Dowlatshahi and Paykel, 1990; Marks et al., 1992; Perry et al., 2019, 2016). One factor potentially involved in the aetiology of PP is the disruption to sleep that is so common in the perinatal period. This hypothesis is based on several lines of reasoning. First, sleep loss, or more specifically a reduced need for sleep, is experienced by most individuals during episodes of mania (66-99%; Steardo et al. 2019). Moreover, sleep loss has been identified as a precipitant of mania in BD in general, with women, specifically those with BD-I, being particularly likely to report this as a triggering factor (Lewis et al., 2017). Third, sleep disruption associated with caring for a new baby occurs almost universally in women during the immediate postpartum (Yang et al., 2020), coinciding with the peak time of onset of symptoms of PP (Langan Martin et al., 2016). Lastly, neurotransmitters such as dopamine and serotonin that are well evidenced to play a role in severe psychiatric disorders, may also interact and overlap to regulate circadian rhythms in relation to both the sleep-wake cycle (Radwan et al., 2019) and hormonal functioning (Gotlieb et al., 2018).

Despite being a promising avenue for futher research, few studies have examined the role of sleep disruption in relation to PP. In a large sample of parous women with BD who were assessed retrospectively, we have previously shown those who report sleep loss as a lifetime trigger of manic episodes to be approximately twice as likely to have a history of PP compared to women who did not report this (Lewis et al., 2018). However, we were unable to assess whether sleep loss within pregnancy or the early postpartum period was associated with subsequent risk of PP within the same perinatal period. To our knowledge, only two published studies have attempted to address this specific question (Bilszta et al., 2010; Sharma et al., 2004). In the first, Sharma, Smith and Khan (2004) used a range of indirect measures to compare sleep disruption occurring during labour between women who experienced PP and a healthy control group recruited from the same hospital matched for age, parity and year of hospital admission. The authors found that compared to the control group, women with PP were significantly more likely to report a longer duration of labour and to have given birth in the night-time, suggesting these women may have experienced more sustained sleep loss in relation to labour and delivery. In the second study, Bilszta et al (2010) gathered detailed data on sleep patterns longitudinally throughout the perinatal period via comprehensive sleep diaries, and compared these between pregnant women at ‘high risk’ of PP (defined as women with BD or a history of PP) and a healthy pregnant control group (Bilszta et al., 2010). While no differences were found between the two groups in overall sleep patterns during pregnancy or the postpartum, sleep disruption in relation to labour and delivery was not assessed. Moreover, due to limitations of sample size, the association between perinatal sleep patterns and PP occurring within the same perinatal period could not be examined.

In light of previous literature, the current study therefore aimed to assess whether perinatal sleep disruption is associated with the occurrence of PP in a sample of pregnant women with BD and therefore at high risk of PP, recruited to a prospective, longitudinal study. Objective measures of sleep such as laboratory based polysomnography and actigraphy are the gold standard methodology, however, feasibility and transferability of these methods is reduced in non-laboratory settings (Van De Water et al., 2011). As these issues are especially pertinent within the perinatal period, we used a range of bespoke, alternative measures to assess sleep disruption occurring in relation to pregnancy and childbirth, with a view to examine the utility of these for future research. To further investigate specificity of the potential relationship between sleep disruption and PP, we also assessed whether perinatal sleep disruption was associated with episodes of postpartum non-psychotic depression.

## Methods

### Participants

Participants were recruited as part of the UK Bipolar Disorder Research Network Pregnancy Study (BDRN.org), an ongoing study which aims to assess a wide range of potential risk factors (including perinatal sleep disruption) for postpartum recurrence of BD. The research programme has NHS Health Research Authority (HRA) approval (Research Ethics Committee (REC) reference MREC/97/7/01) and Research and Development approval in all participating NHS Trusts/Health Boards. Women were recruited systematically via UK National Health Service (NHS) psychiatric services or non-systematically via the BDRN website or national patient support charities, Bipolar UK and Action on Postpartum Psychosis. Women are eligible to participate in the BDRN Pregnancy Study if they are i) aged 18 years or over, ii) meet DSM-5 criteria for BD and iii) are currently at or beyond 12 weeks gestation of pregnancy. Women are excluded if they have only ever experienced mood illness secondary to substance use or medical illness or were unable to provide written informed consent.

The sample in the current study comprised 76 women with BD-I (n=74) or schizoaffective disorder bipolar type (SA-BD; n=2), who were not in a mood episode at the time of delivery and from whom information about sleep during the perinatal period had been obtained.

### Procedure

This study utilised a prospective, longitudinal design. Women participated in the study from 12 weeks of pregnancy (baseline) to three months postpartum. A baseline semi-structured interview (Schedules for Clinical Assessment in Neuropsychiatry; SCAN (Wing et al., 1990)) was conducted in person or via telephone during pregnancy to assess demographic factors, parity and lifetime psychopathology before pregnancy (including best-estimate main lifetime diagnosis at the time of current pregnancy according to DSM-5 criteria). Information about potential risk factors for recurrence (including sleep disruption), medication use and the presence or absence of psychopathology within the current perinatal period were assessed by additional interviews (also comprising the SCAN) administered by telephone in the third trimester of pregnancy and at three months postpartum. All assessments were conducted by a trained research psychologist or psychiatrist. Women recruited to the study from 24 weeks of pregnancy completed baseline and third trimester assessments simultaneously. For women who provided written consent for clinician contact, a postal questionnaire was completed by their psychiatrist and/or general practitioner at two months postpartum (n=64). Information was obtained from clinician questionnaires regarding the outcome of pregnancy (to ensure we were aware of the outcome prior to contacting women for follow-up at three months postpartum) and any episodes of mood illness that had occurred within the perinatal period. Where consent was provided, assessment data were supplemented by information obtained from psychiatric case-notes (n=58). Ratings were made from detailed written vignettes compiled from all available data. All ratings were made independently by at least two members of the study team and consensus agreed by discussion.

### Assessment of sleep in relation to pregnancy and childbirth

- A question from the Pittsburgh Sleep Quality Index (PSQI; Buysse et al. 1989) was used to assess women’s subjective experience of sleep quality reported in the third trimester of pregnancy (at median 33 weeks of pregnancy, range 24-39). The PSQI is a self-report instrument assessing sleep quality and disturbance over a one-month period and has been widely used in a perinatal population (González-Mesa et al., 2019). Women were asked ‘during the last month, how would you rate your sleep quality overall?’, with possible responses coded as ‘very good’, ‘fairly good’, ‘fairly bad’ and ‘very bad’. For the purpose of this study, responses were collapsed to ‘very good/fairly good’ and ‘fairly bad/very bad’.
- Information about sleep loss in relation to labour/delivery was obtained at the three-month postpartum follow up interview. To be consistent with previous literature (Sharma et al., 2004), women were asked the length of their labour (in hours) and the time of delivery of their infant as measures of sleep loss in relation to labour/delivery. Time of delivery was coded categorically as day time (07:31am-12:29am) or night time (12:30am-07:30am). In the absence of standardised measures, a bespoke question was also asked at postpartum follow-up as a more direct measure of sleep loss in relation to labour/delivery. Specifically, women were asked if they had lost any sleep in relation to labour/delivery and the number of complete nights of sleep missed (defined as the number of complete nights without any sleep occurring between the onset of labour [time of first contraction] and the first complete night of sleep). Responses were coded categorically as ‘no complete nights of sleep missed’ or ‘one or more complete nights of sleep missed’.

### Postpartum psychiatric outcomes

The most impairing DSM-5 episode of psychiatric illness with onset within 12 weeks postpartum was rated for each woman. In cases in which an episode of mania or psychosis and an episode of any other type of affective illness (for example, mania/psychosis and major depression without psychosis) had occurred with onset within 12 weeks of delivery, the episode of mania or psychosis was rated hierarchically as the most impairing episode. Patterns of perinatal psychiatric outcomes within the BDRN pregnancy sample have been reported and discussed in detail previously (Perry et al., 2021a). Within the current study, 33/76 (43.4%) women experienced the onset of at least one DSM-5 mood episode within 12 weeks of childbirth. Ratings of the most impairing mood episode with onset in the postpartum period were used to define the following outcome groups:

- PP within six weeks of delivery (n=20): mania or psychosis (including depression with psychotic features) with onset within six weeks of childbirth. A temporal cut-off of six weeks was used to define the onset of PP to be consistent with previous research (Perry et al., 2021a) and to capture both DSM-5 and ICD-10 definitions of the postpartum period. Of the 20 women included within this group, 11 experienced an episode of mania with psychotic features, eight mania without psychotic features and one an episode of depression with psychotic features. No women experienced an episode of non-affective psychosis.
- PPD within 12 weeks of delivery but no PP (n=10): major depression without psychosis with onset within 12 weeks of childbirth. A broader temporal cut-off was used to define the onset of PPD, due to the onset of these episodes being more widely distributed across the postpartum period (Langan Martin et al., 2016).
- No PP or PPD within 12 weeks of delivery (n=46); defined as women who experienced no DSM-5 mood episode (n=43) and those who experienced an episode of hypomania (n=3).

### Prophylactic mood stabilising medication use in the postpartum period

Prophylactic mood stabilising medication in the postpartum period was defined as use of lithium, anticonvulsants or antipsychotics [atypical or typical] commenced or continued within the postpartum period for reasons other than treatment. Consistent with this definition, 44/76 (58%) women utilised mood stabilising medication prophylactically in the postpartum period, comprised of those who used lithium only (n=2), antipsychotics only (n=21), lithium in combination with antipsychotics (n=3), anticonvulsants in combination with antipsychotics (n=2) and use of at least one mood stabiliser in combination with an antidepressant (n=16).

### Statistical analysis

Data were analysed using SPSS version 28. Perinatal sleep factors were described according to postpartum psychiatric outcome, and compared using chi-squared or Fisher’s exact tests. Potential confounding factors were assessed for inclusion in multivariate analyses by comparing postpartum outcome groups for demographics, within-pregnancy and key psychiatric history characteristics at the time of the current pregnancy using Mann-Whitney U, chi-squared and Fisher’s exact tests. Associations between perinatal sleep factors and postpartum psychiatric outcomes that were significant in univariate analysis were subsequently assessed in a logistic regression model adjusted for the use of prophylactic mood stabilsing medication in the postpartum period. Odds ratios (OR) are presented with 95% confidence intervals (CI). P values of <0.05 were considered statistically significant.

## Results

### Subjective sleep quality in late pregnancy and postpartum psychiatric outcome

No significant association was found between self-reported sleep quality in the third trimester of pregnancy and postpartum psychiatric outcome (p=0.412; see Table 1). This analysis was repeated including only those women who reported on sleep quality within the final four weeks prior to childbirth (n=26) to ensure that sleep quality was measured as close to delivery as possible, and there remained no significant association between sleep quality and postpartum psychiatric outcome (p=0.320).

### Sleep disruption associated with labour/delivery and postpartum psychiatric outcome

**Figure 1:**
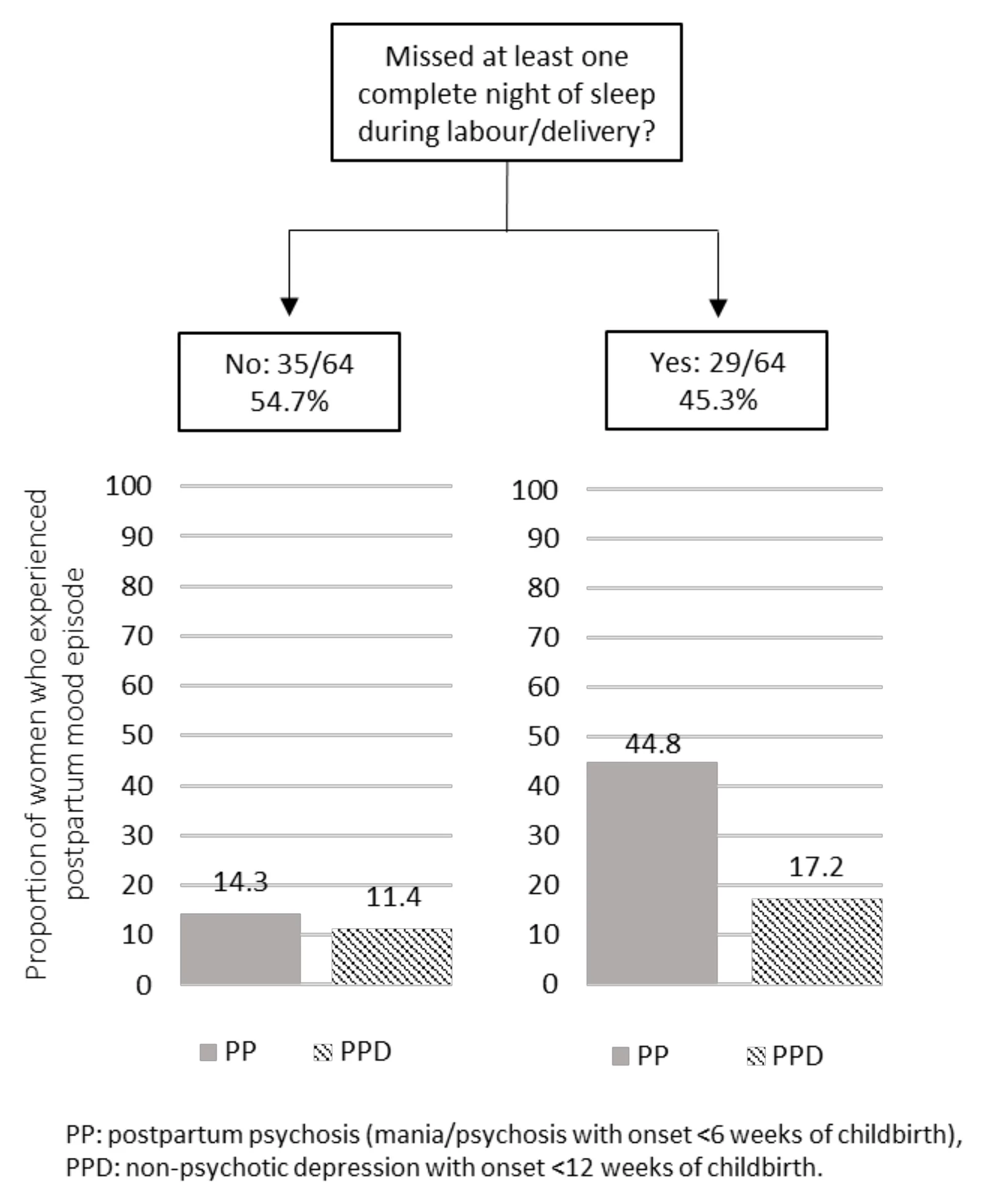
Flow chart showing proportion of women with BD-I/SA-BD who experienced a postpartum mood episode according to presence/absence of at least one complete night of missed sleep in relation to labour/delivery

No significant association was found between length of labour or time of delivery and postpartum psychiatric outcome (p=0.158 and p=0.422 respectively). As shown in Figure 1, women who reported missing at least one complete night of sleep in relation to labour/delivery were significantly more likely to experience PP compared to women who did not miss any complete nights of sleep (44.8% v 14.3%; 95% CI 8.4-52.7%, p=0.003). Though episodes of PND also occurred more frequently in women who missed at least one complete night of sleep in relation to labour/delivery compared to those who did not, this was not statistically significant (17.2% v 11.4%, 95% CI -11.3-22.9%; p=0.241).

### Demographic, within-pregnancy and psychiatric history characteristics at the time of pregnancy

No significant differences were found between postpartum outcome groups in relation to demographic, within-pregnancy specific or psychiatric history characteristics at the time of the current pregnancy (Table 2).

### Relationship between perinatal sleep factors and postpartum psychiatric outcome after adjusting for covariates

After adjusting for use of prophylactic mood stabilising medication in the postpartum period, women who reported the loss of at least one complete night of sleep across labour/delivery were at significantly greater odds of experiencing PP compared to women who did not report this (OR 5.19, 95% CI 1.45-18.54, p=0.011).

### Sleep disruption as a potential trigger of postpartum psychosis

To further assess the role of sleep disruption in relation to labour/delivery as a potential trigger of PP, we examined time of onset of PP in women who reported missing one or more nights sleep across labour/delivery and those who did not report this. Distribution of times of onset of episodes of PP clustered more closely to delivery among those who reported one or more missed night of sleep compared to those who did not miss any sleep. Specifically, all episodes of PP among those who reported one or more missed nights of sleep in relation to labour/delivery had onset within the first 3 weeks postpartum, with 92% episodes having onset within the first two weeks following delivery. In contrast, episodes of PP among women who did not report sleep loss in relation to labour/delivery were more widely distributed across the postpartum period, with onset occurring up to 5 weeks postpartum and 60% of episodes occurring within the first two weeks after delivery.

## Discussion

To our knowledge, this study is the first to longitudinally examine the association between perinatal sleep disruption and the occurrence of PP in a well characterised sample of pregnant women with BD. We found that women with BD who experienced the loss of at least one complete night of sleep in relation to labour and/or delivery had over five times the odds of experiencing PP compared to women with BD who experienced no or less than one night of sleep loss. This relationship remained significant after controlling for use of prophylactic mood stabilising medication in the postpartum period. Conversely, no association was found between any other perinatal sleep factor assessed (self-reported poor sleep quality in pregnancy, length of labour and time of delivery) and PP, nor between any perinatal sleep factor and the occurrence of PPD.

Our findings are consistent with those of studies in which sleep loss has been implicated as a trigger of non-puerperal episodes of mania (Lewis et al., 2017) as well as those that have onset following childbirth (Lewis et al., 2018; Sharma et al., 2004). Previously, we demonstrated a lifetime vulnerability to mania triggered by sleep loss to be associated with PP in women with BD (Lewis et al., 2018). Here however, using contemporaneous assessments, we were able to extend these findings further, specifically examining the association between sleep disruption and PP occurring within the same perinatal period.

Interestingly, despite our finding that self reported sleep loss in relation to labour/delivery (a more direct measure of sleep loss) was associated with PP, in contrast to Sharma et al. (2004), we found no such association between length of labour or time of delivery and PP in our sample. Accounting for this difference, it is possible that length of labour and time of delivery do not correlate accurately with amount of sleep lost in relation to labour/delivery. Indeed, in our sample, women who reported missing at least one complete night of sleep in relation to labour/delivery were not significantly more likely to experience a night-time delivery compared to those who did not miss sleep (17.4 and 14.7%, p=1.000). Moreover, in our study, data were gathered via participant interviews conducted in the postpartum period, compared to data collected by medical case note review by Sharma et al (2004). In addition, Sharma et al (2004) utilised a control group comprised of parous women without a history of psychiatric illness, while ours consisted of parous women with BD-I who did not experience an episode of PP; women who were also at high risk of experiencing of PP.

In contrast to our finding that acute severe sleep deprivation occurring in relation to childbirth was associated with PP, we did not find an association between subjectively reported poor quality of sleep in the third trimester of pregnancy and PP. Though not directly comparable, this finding is supported by that observed by Bilszta, Meyer and Buist (Bilszta et al., 2010), who reported no significant differences in sleep patterns during pregnancy in women at high risk of PP and a healthy pregnant control group. Together, this may suggest that childbirth related factors, rather than pregnancy related factors are particularly important in the pathogenesis of PP. On the whole, this is consistent with evidence which clearly indicates the immediate postpartum period, rather than pregnancy to be a time of particularly high risk of severe mood episodes in women with BD (Munk-Olsen et al., 2009). Combined with previous research, these findings therefore suggest that in the context of other important childbirth related factors implicated in the aetiology of PP (such as hormonal, immunological and obstetric factors), vulnerability to acute and severe sleep deprivation occurring during childbirth or the immediate postpartum period may act as a final trigger of PP.

Unlike our findings in relation to PP, we found no significant association between any perinatal sleep factor assessed and the occurrence of PPD, suggesting specificity of the sleep loss trigger to PP in women with BD. This finding is inconsistent with studies conducted within the general population and in samples of women with unipolar major depression, in which chronic sleep disruption during the perinatal period has been associated with the presence and severity of postpartum depressive symptoms (Dørheim et al., 2014). Several possible reasons may explain this disparity in findings. For example, evidence suggests that bipolar postpartum depression may be distinct from other forms of depression occurring following childbirth (Sharma et al., 2017). Thus, the mechanisms involved in the aetiology and triggering of PPD in BD may also be distinct. Moreover, we defined episodes of PPD according to standardised diagnostic criteria (DSM-5), while previous studies have predominantly defined outcome based on symptom scores of depression assessed using brief psychometric screening tools (Dørheim et al., 2014). In addition, we only assessed sleep disruption occurring within a one-month period in the third trimester of pregnancy and across labour and delivery. It remains possible that more chronic and sustained sleep loss that occurs over a longer period of time (for example, in relation to breastfeeding postpartum) is associated with episodes of PPD, and in particular, with episodes that have onset beyond three months postpartum. Though we were not able to assess this hypothesis in the current study, we have previously shown that women with BD who report sleep loss as a frequent trigger of depressive episodes, were not significantly more likely to experience PPD within six weeks or six months of childbirth when assessed retrospectively (Lewis et al., 2018).

## Limitations

Our study is subject to several limitations. First, we were not able to objectively measure sleep disruption during the perinatal period. In particular, sleep loss occurring in relation to labour and delivery was assessed retrospectively via self-report at three months postpartum and is therefore potentially vulnerable to recall error and bias. Research has however demonstrated women’s recall of events related to labour and delivery to be excellent when assessed weeks (Troude et al., 2008) and even months postpartum (Bat-Erdene et al., 2013) and our data was also supplemented by clinician reports and contemporaneous psychiatric case-notes. Second, women with BD-II or other specified bipolar disorders were not included in this study and generalisability of our findings may therefore be restricted to those with BD-I and SA-BD only. Similarly, we defined the postpartum period as the first 12 weeks following childbirth and episodes of illness that have onset beyond this time frame would not have been captured by this definition. For this reason, our findings may also not extend to episodes of mood illness that have onset later in the postpartum period, in particular, episodes of non-psychotic depression. Finally, it was not possible in our study to distinguish whether sleep loss occurring in relation to childbirth was a prodromal symptom of PP, or a triggering factor. Notably, women themselves did not attribute this form of sleep loss to the early onset of PP, but rather to the circumstances related to labour and delivery. Moreover, we found that timing of onset of PP among women who reported sleep loss in relation to labour/delivery was more closely clustered around the time of childbirth compared to those who did not report sleep loss. Together, this might suggest a triggering role for acute sleep loss in the onset of PP episodes. To examine the precise nature and direction of this relationship further, prospective studies of pregnant women with BD that include objective measures of sleep within the perinatal period and in relation to labour and delivery are required. Nonetheless, irrespective of the cause, our finding arguably suggests that acute sleep deprivation occurring in relation to childbirth and the immediate postpartum may remain a useful marker of risk of PP, providing an important opportunity for early intervention and prevention in women with BD.

## Conclusions

In conclusion, we found evidence to suggest that in women at high risk of PP due to a past history of BD, sleep deprivation equivalent to missing at least one complete night of sleep at the time of delivery/in the immediate postpartum is an important marker of risk of PP. Our results indicate a large (OR=5.2) and potentially clinically significant effect. To investigate the nature of this relationship further, prospective studies of pregnant women with BD should ideally aim to measure sleep in the perinatal period and in relation to childbirth using objective methods, such as actigraphy. However, should this not be feasible, we have identified an alternative simple and efficient measure of perinatal sleep disruption that we have demonstrated to predict risk of PP that can be employed within future research. If replicated, our findings would not only contribute to understanding of the aetiology of mood disorders more generally, but importantly, have clinical implications for the management of women at high risk of PP in the perinatal period. In particular, this may include consideration of potential interventions to minimise sleep disruption in relation to childbirth and also to feeding (especially at night) in the immediate postpartum as far as possible.

## Figures and Tables

**Table 1:** Perinatal sleep factors according to postpartum psychiatric outcome

	PP	PPD only	No PP/PPD	p-value	Post-hoc comparison
**Sleep factor**					
**Sleep quality in 3^rd^ trimester**				0.412†	-
Good/very good (n=30)	30% (9)	6.7% (2)	63.3% (19)		
Poor/very poor (n=33)	24.2% (8)	18.2% (6)	57.6% (19)		
**Length of labour (hours)**				0.158	-
Median	9	18	6		
IQR (range)	24 (0-96)	29 (0-48)	12 (0-48)		
**Time of delivery**				0.422	-
Day time (07:31am-12.29am, n=50)	28% (14)	12% (6)	60% (30)		
Night time (12:30am-07:30am, n=10)	10% (1)	20% (2)	70% (7)		
**At least one complete night’s sleep lost** **in relation to labour/delivery**				0.009** ^†^	PP vs PPD; p=0.423^‡^ **PP vs No PP/PPD; p=0.003 ^‡^** ** PPD vs No PP/PPD; p= 0.241 ^‡^
No complete night lost (n=35)	14.3% (5)	11.4% (4)	74.3% (26)		
At least one night lost (n=29)	44.8% (13)	17.2% (5)	37.9% (11)		

Ns differ due to unknown data. PP: postpartum psychosis (mania or psychosis) with onset within six weeks of delivery. PPD: non-psychotic depression (but not PP) with onset within three months postpartum. No PP/PPD: no postpartum psychosis or postpartum depression within three months of delivery. ^†^Fisher’s exact test. ‡ Chi-square test *p<0.05 **p<0.01

**Table 2:** Demographics, parity and clinical characteristics according to postpartum psychiatric outcome.

	PP N=20	PPD N=10	No PP or PPD N=46	p-value
**Demographics at time of current pregnancy**				
**Age (years) at current pregnancy**				
Median (IQR)	34 (9)	33 (6)	34.5 (6)	0.678
Range	20-44	24-40	24-43	
**Ethnicity**				
White	90.0% (18)	90.0% (9)	95.7% (44)	0.503
Non-white	10.0% (2)	10.0% (1)	4.3% (2)	
**Recruitment**				
Systematic	50.0% (10)	70.0% (7)	54.3% (25)	0.640
Non-systematic	50.0% (10)	30.0% (3)	45.7% (21)	
**Education**				
Degree	60.0% (12)	70.0% (7)	67.4% (31)	0.780
No degree	40.0% (8)	30.0% (3)	32.6% (15)	
**Factors specific within current pregnancy**				
**Parity**				
Primiparous	40.0% (8)	70.0% (7)	45.7% (21)	0.305
Parous	60.0% (12)	30.0% (3)	54.3% (25)	
**Planned pregnancy**				
Yes	68.4% (13)	66.7% (6)	85.7% (36)	0.162
No	31.6% (6)	33.3% (3)	14.3% (6)	
**Obstetric complications in pregnancy^+^**				
Yes	26.3% (5)	50.0% (5)	47.6% (20)	0.253
No	73.7% (14)	50.0% (5)	52.4% (22)	
**Obstetric complications in delivery ^+^**				
Yes	42.1% (8)	77.8% (7)	55.0% (22)	0.222
No	57.9% (11)	22.2% (2)	45.0% (18)	
**Prophylactic mood stabiliser use in postpartum**				
Yes	55.6% (10)	70% (7)	58.7% (27)	0.746
No	44.4% (8)	30% (3)	41.3% (19)	
**Psychiatric history at time of current pregnancy**				
**Age (years) at onset of impairing BD**				
Median (IQR)	21 (9)	18 (5)	20 (10)	0.210
Range	13-36	15-23	11-36	
**Number of episodes of mania per illness year (avg.)**				
Median (IQR)	0.29 (0.33)	0.67 (2.35)	0.33 (0.43)	0.211
Range	0.11-2.01	0.08-3.42	0.05-7.37	
**Number of episodes of depression per illness year (avg.)**				
Median (IQR)	0.40 (0.79)	0.51 (0.63)	0.44 (0.37)	0.723
Range	0-1.85	0.06-1.11	0-4.34	

Ns differ due to unknown data. PP: postpartum psychosis (mania or psychosis) with onset within six weeks of delivery. PPD: non-psychotic depression (but not PP) with onset within three months postpartum. No PP/PPD: no postpartum psychosis or postpartum depression within three months of delivery. ^†^ Obstetric complications defined according to ICD-10 criteria (World Health Organisation, 2012)

## Data Availability

The data that support the findings of this study are available on reasonable request from the corresponding author. The data are not publicly available due to privacy or ethical restrictions.

## Highlights

- Perinatal sleep factors and postpartum psychiatric outcomes assessed longitudinally
- Acute, childbirth related sleep loss associated with postpartum psychosis
- No relationship between perinatal sleep factors and postpartum depression
- Implications for risk prediction of postpartum psychosis in bipolar disorder
